# Intranasal Mucoadhesive In Situ Gel of Glibenclamide-Loaded Bilosomes for Enhanced Therapeutic Drug Delivery to the Brain

**DOI:** 10.3390/pharmaceutics17020193

**Published:** 2025-02-04

**Authors:** Meenakshi Tripathi, Laxmi Gharti, Amit Bansal, Hemlata Kaurav, Sandeep Sheth

**Affiliations:** 1 School of Pharmaceutical Sciences, Shoolini University of Biotechnology and Management Sciences, Solan 173229, Himachal Pradesh, India; meenakshitripathi15012000@gmail.com (M.T.); lakshmi053gharti@gmail.com (L.G.); 2 Formulation Research and Development, Perrigo Company plc, Allegan, MI 49010, USA; amitbansal2010@gmail.com; 3 Department of Pharmaceutical Sciences, College of Pharmacy, Larkin University, Miami, FL 33169, USA

**Keywords:** glibenclamide, bilosomes, intranasal, in situ gel, brain drug delivery

## Abstract

Background: The neuroprotective efficacy of glibenclamide (GLIB) has been demonstrated in multiple rodent models of ischemia, hemorrhagic stroke, traumatic brain damage, spinal cord injury, and metastatic brain tumors. Due to its poor solubility, GLIB has low oral bioavailability, limiting its transportation to the brain via the oral route. Objectives: Here, we attempted to develop and optimize an intranasal mucoadhesive in situ gel of GLIB-loaded bilosomes using a 3^2^ Box–Behnken design for brain drug delivery. Methods: To facilitate a longer residence time of the administered dose within the nasal cavity, the prepared bilosomes were loaded into a mucoadhesive in situ gel providing resistance to rapid mucociliary clearance. The amounts of sodium deoxycholate, the cholesterol/Span 40 mixture, and the molar ratio between the mixture’s components were chosen as independent variables, while the entrapment efficiency and in vitro drug release were selected as dependent variables. Results and conclusions: The optimal formulation was analyzed for particle size and entrapment efficiency, which were found to be 270.6 nm and 68.39%, respectively. In vitro drug release from optimal formulation after 12 h was 87.29 ± 1.98% as compared to 52.01 ± 2.04% of plain in situ gel of drug. An in vivo brain drug delivery study performed on Swiss albino mice showed that the brain concentration of drug through intranasal administration from mucoadhesive in situ gel of GLIB-bilosomes after 12 h was 2.12 ± 0.16 µg/mL as compared to 0.68 ± 0.04 µg/mL from plain in situ gel of drug. Conclusively, the developed bilosomal formulation offers a favorable intranasal substitute with enhanced therapeutic drug delivery to the brain.

## 1. Introduction

The central nervous system (CNS) a complex system within the human body. Effective barriers to the passage of medicines from the bloodstream into the CNS are the blood–brain barrier (BBB) and the blood–cerebrospinal fluid barrier (BCB), particularly for polar and large-molecular-weight medications like peptides and proteins [1]. Since the number of elderly people and patients with CNS problems is rising, there will be an increase in the worldwide drug development market for brain diseases during the next 20 years. Compared to other therapeutic possibilities, the development of medications for brain illnesses has the lowest success rates, and the time required to develop drugs for the treatment and management of CNS disorders is typically significantly greater than that of other drug types [2]. For the past few decades, parenteral and oral routes have been considered as preferable routes of administration because of their convenience, increased patient compliance, and cost effectiveness. The treatment of neurological disorders mainly includes the administration of therapeutic agents via topical and intravenous routes, and device-based therapies like deep brain stimulation, surgeries, and rehabilitation are also used. Other drug delivery advances include the direct CNS delivery of drugs via injection into the cerebrospinal fluid and intranasal delivery. Some of these methods have the drawbacks of being invasive, perilous, local, and transient. Therefore, to overcome these issues, the administration of therapeutic agents via the nose-to-brain route can be explored. The nasal mucosa has become a valuable target tissue for brain medication delivery due to its high vascularity, vast surface area, porous endothelial membrane, accessibility, and resistance to hepatic first-pass metabolism when compared to the oral route of drug administration. Moreover, the nasal passage lacks pancreatic and stomach enzymatic activity, due to which compounds are more permeable via the intranasal route than the gastrointestinal tract. Intranasal drug delivery also permits dosage reduction with a shorter half-life, a quicker start to pharmacological activity, and a reduced incidence of side effects [3]. For hydrophobic drugs, having a short half-life requires frequent dosing to maintain therapeutic levels that can lead to patient non-compliance and increased risk of adverse effects due to higher cumulative doses. This situation is exacerbated by their inherent low solubility, necessitating higher doses to achieve desired plasma concentrations. Incorporating drugs within the bilosome system leads to prolonged drug release, leading to dose reduction with safer and effective therapeutic outcomes [4].

Glibenclamide (GLIB), also referred to as glyburide, is mostly prescribed orally as an anti-diabetic drug to treat type 2 diabetes. But interest in its possible neuroprotective benefits is growing, especially when it comes to neurological diseases like traumatic brain injury (TBI) and stroke. Sulfonylurea receptor 1 (SUR1)-regulated NC_Ca-ATP_ channels are nonselective cation channels that are believed to contribute to brain edema and neuronal damage after stroke and TBI and are blocked by GLIB. GLIB has been revealed to reduce inflammation and oxidative stress in the brain, which is a key contributor to neuronal damage in various neurological conditions [5,6,7]. Very few formulations of GLIB are reported in the scientific literature for treating neurological conditions, mainly osmotic pumps [8] and in situ forming microparticles [5]. As per recent reports, glyburide for injection (RP-1127, BIIB093) is under clinical trial (NCT01132703) (https://clinicaltrials.gov) (assessed on 19 May 2024) for the treatment and prevention of severe cerebral edema in neurological disorders [9,10]. Currently, no marketed formulation of GLIB is approved by the FDA for neuroprotection.

Bilosomes are bilayer structures of non-ionic amphiphiles that closely correlate to non-ionic surfactant vesicles but incorporate bile salts into them. Bile acids have been documented as biosurfactants with major roles in endogenous organotropism and have been frequently utilized as penetration enhancers by pharmaceutical manufacturers for years. One of the advantages of bilosomes is their ultra-deformability and stability, which increase drug absorption and bioavailability. Bilosomes can be engineered for targeted drug delivery to specific cells or regions of the brain, leading to increased drug delivery [11,12]. GLIB is a poorly soluble compound with very low oral bioavailability (45%) [13], which severely limits its transportation to the brain via the oral route. Moreover, it suffers from hepatic first-pass metabolism. To improve GLIB solubility and BBB permeability and mitigate first-pass metabolism, the current study was focused on developing intranasal in situ gel of GLIB-loaded bilosomes (BLs). The novelty of our study lies in developing an optimized intranasal mucoadhesive in situ gel formulation of GLIB-loaded bilosomes, which enhances brain drug delivery while overcoming the limitations of poor oral bioavailability and frequent dosing. Conventional treatments for neurological disorders often rely on oral administration, which results in low drug bioavailability due to the constraint of blood–brain barrier and poor solubility of many drugs. In contrast, the intranasal route offers a more direct pathway to the brain, bypassing the blood–brain barrier and enabling higher concentrations of the drug in the target tissue. To facilitate longer residence of the administered dosage form within the nasal cavity, the developed intranasal bilosomes were loaded into a mucoadhesive in situ gel providing resistance to rapid mucociliary clearance. We investigated selected formulation variables using the 3^2^ Box–Behnken design for brain drug delivery. This work is thought to be the first to assess GLIB’s brain targeting via intranasal bilosomes crossing the olfactory mucosa.

## 2. Materials and Methods

### 2.1. Drugs and Reagents

GLIB was kindly gifted from Syskem Pharmocrats, Solan, India All other chemicals employed in the current study, such as cholesterol (CHL), Span 40, and sodium deoxycholate (SDC), were obtained from Loba Chemie Pvt. Ltd., Mumbai, India, and were of analytical grade.

### 2.2. Drug–Excipient Compatibility Study Using FTIR Spectroscopy

The drug–excipient compatibility study of GLIB in bilosomal formulations was inspected using Fourier transform infrared (FTIR) spectroscopy (Agilent Technologies, Santa Clara, CA, USA) as described previously [14]. The FTIR spectra of pure GLIB, the individual bilosomal excipients (Span 40, CHL, and SDC), and the physical mixtures of GLIB with the excipients were acquired in the 400–4000 cm^−1^ frequency range, and they were compared to determine any changes occurring to the drug upon being formulated into the nanovesicle system.

### 2.3. Formulation of GLIB-Loaded Bilosomes

GLIB-loaded bilosomes were formulated utilizing the already reported thin film hydration method [15,16]. Briefly, the required dose of the drug (3 mg) was blended with the mixture of cholesterol and Span 40 (non-ionic surfactant) in a varying ratio of 1:1 to 1:9, weighing 100–300 mg and dispersed in chloroform (10 mL) along with bile salt (SDC 5–25 mg) in a round-bottom flask. The organic solvent was evaporated at 60 °C under vacuum using a rotary evaporator set to 90 rpm for 30 min. Afterward, distilled water was added to completely hydrate the thin, dry layer that had formed on the flask’s inner wall. The hydration process used a rotary evaporator spinning at 90 rpm for 1 h at 60 °C while under standard pressure and with glass beads present. Thereafter, the hydrated film dispersion was stored at 6 °C for the entire night after being sonicated for 5 min using a bath sonicator to allow the formation of a homogenous milky dispersion devoid of aggregates [17].

### 2.4. Optimization of GLIB-Loaded Bilosomes

To investigate the impacts of formulation parameters on the primary properties of the manufactured bilosomes and optimize their design, a three-factor, two-level (3^2^) Box–Behnken design was set up, for which Design-Expert^®^ software version 13 (Stat-Ease, Inc., Minneapolis, United States) was utilized. The independent factors comprised the amount of bile salt, i.e., SDC (X_1_), the molar ratio of cholesterol: Span 40 (X_2_), and the amount of cholesterol/Span 40 mixture (X_3_) (Table 1). The entrapment efficiency (Y_1_) and in vitro drug release efficiency at 12 h (Y_2_) were chosen as the dependent factors or variables and preferred to be in range. The range set for response entrapment efficiency was 40–70% and for in vitro release efficiency was 80–100%. According to the Box–Behnken design, 17 formulations were suggested and prepared as presented in Table 2.

ANOVA and multiple linear response analysis were used to derive the following equation for each response variable. The design was estimated by the quadratic model using the following equation [5]:

Y = A_0_ + A_1_X_1_ + A_2_X_2_ + A_3_X_3_ + A_4_X_1_X_2_ + A_5_X_1_X_3_ + A_6_X_2_X_3_ + A_7_X_1_^2^ + A_8_X_2_^2^ + A_9_X_3_^2^ (1)

where Y is the measured response variable; A_0_ is the constant; and A_1_, A_2_, A_3_, A_4_, A_5_, A_6_, A_7_, A_8_, and A_9_ are the regression coefficients. X_1_, X_2_, and X_3_ are the main effects; X_1_X_2_, X_2_X_3_, and X_1_X_3_ represent the interaction terms and reflect the change in response when three factors are simultaneously altered; and Ai^2^ (i = 1, 2 or 3) signify the quadratic terms individually. To identify the optimized formulation, a numerical and graphical optimized procedure with a desirability approach was utilized. To authenticate the response, the optimal formulation was prepared and investigated for percent entrapment efficiency and in vitro release efficiency at 12 h as forecasted using the optimization methodology. Percentage prediction error was estimated by comparing the predicted and experimental values [18]. It was calculated as follows:

(Experimental value − Predicted value)/Experimental value × 100(2)

### 2.5. In Vitro Evaluation of Developed Formulation—Entrapment Efficiency (EE%)

Bilosome dispersion (1 mL) was combined with 9 mL of ethanol and sonicated for 5 min using a bath sonicator to evaluate the EE%. The actual drug content was measured using HPLC by measuring its absorbance at a wavelength of 230 nm, following an appropriate dilution. Furthermore, a cooling centrifuge was used to ultra-centrifuge the same volume of the bilosomal formulation for 60 min at 15,000 rpm and 4 °C. To measure the amount of drug entrapped, the supernatant was discarded, and the residue was dissolved in 10 mL of ethanol under sonication for 5 min. The GLIB content was assayed using HPLC [16,17]. EE% was determined as follows:

EE% = (Amount of drug entrapped/Actual amount of drug) × 100(3)

### 2.6. In Vitro Drug Release Study

The dialysis bag approach was used, in which a dialysis bag that had been pre-soaked for 12 h in distilled water was filled with 0.2 mL of bilosome dispersion carrying an equivalent quantity of dose. The filled dialysis bag was tightly fastened on both ends and then plunged into 50 mL of dissolution media consisting of phosphate-buffer saline (PBS) (pH 7.4) and ethanol in the ratio of 80:20 in a conical flask to ensure the sink condition. Ethanol was utilized to enhance the solubility of poorly soluble drug in the dissolution media. Then, the conical flask was positioned in a thermostatically controlled shaking water bath running at 100 shakes per minute at 37 ± 0.5 °C. 1 mL sample of the aliquots were withdrawn at specific time intervals (1, 3, 4, 6, 8, and 12 h) and instantly replaced with equivalent volumes of fresh PBS (pH 7.4). Samples were assayed using HPLC for GLIB content at λ_max_ 230 nm [17,19].

### 2.7. Particle Size, Polydispersity Index (PDI), and Zeta-Potential

Dynamic light scattering was used to analyze the globule size and measure the PDI. The optimal formulations were sonicated and then evaluated for globule size and PDI assessment after being diluted 50 times with distilled water. The zeta potential of the optimal bilosome system was determined using a Litesizer DLS 500 zetasizer (Anton-Paar, Graz, Austria) [20,21].

### 2.8. Transmission Electron Microscopy (TEM)

Morphological examination of the optimal GLIB-loaded bilosomes was performed using TEM (Hitachi H-7500, Hitachi High-Tech Science Corporation, Tokyo, Japan). An hour before the examination, a drop of diluted material was applied to a copper grid coated with perforated carbon film. Then, the sample was visualized and examined using TEM [20,22].

### 2.9. Differential Scanning Calorimetry (DSC)

The thermal properties of the plain drug, excipient mixture, and optimized formulation were examined using differential scanning calorimetry (SETLINE^®^ DSC+, Setaram, KEP Technologies, Geneva, Switzerland). Nitrogen was used to create an inert environment while the heat runs for each sample were designed to range from 100 °C to 250 °C at a rate of 10 °C per minute, containing 3 to 5 mg of product in perforated aluminum sealed pans [23].

### 2.10. Preparation and Evaluation of Mucoadhesive In Situ Gel Preparation of Optimal Bilosomes

A gelling system of optimal GLIB-loaded bilosomes was prepared using the cold method [24]. Mucoadhesive in situ gel was prepared with the addition of accurately weighed 0.4% hydroxy propyl methyl cellulose (HPMC-K4M) into the bilosomal dispersion. The bioadhesive properties of HPMC are primarily attributed to its abundant -OH functional groups, which facilitate hydrogen bonding with water and other HPMC molecules. Furthermore, HPMC exhibits minimal interactions with drugs beyond hydrogen bonding, while effectively enhancing bioadhesion and improving the localized delivery of drugs through increased retention [25]. Poloxamer 407 at a concentration of 17% *w*/*v* was further added gradually under magnetic stirring at 100 rpm and at room temperature. In order to allow the produced dispersion to equilibrate and form a clear solution, it was kept in a refrigerator at 6 °C for 24 h [26,27].

The optimal GLIB-loaded bilosome mucoadhesive in situ gel was further evaluated for pH (using digital pH meter), rheological parameters (flow index and consistency index), sol-to-gel transition temperature, time, and drug release parameters [28].

The tilting approach was employed to ascertain the sol-to-gel transition temperature of the produced gelling system. A test tube was sealed with parafilm and placed in a water bath with a 2 mL aliquot of the prepared clear solution of the mucoadhesive in situ gel inside of it. The water bath temperature was raised gradually, beginning at 20 °C and going up by 0.5 °C every 10 min. Tilting the material by ninety degrees allowed for a visual inspection for gelation. The examined formulation’s sol-to-gel transition temperature was also its gelation temperature [29,30].

The test tube inversion method was used to study the sol-to-gel transition time. To summarize, a 5 mL stoppered test tube containing 1 mL of the mucoadhesive in situ gelling system (sol) was placed in a 37 °C water bath with a thermostat. By tilting the tube every 10 s until no flow was detected, the changeover time was established [31].

The optimized formulation was further subjected to rheological tests to ascertain the flow behavior. An R/S-CPS-P1 rheometer with Rheoplus 32 software was used for this purpose. By using the power law equation, the flow index and consistency index of the optimal gel formulation were calculated from the shear rate vs. shear stress plot [28].

### 2.11. Assessment of Drug Release Parameters of Mucoadhesive In Situ Gel of GLIB-Loaded Bilosomes

As stated earlier under the bilosomes in vitro release tests, the mucoadhesive in situ gelling system loaded with optimum GLIB-loaded bilosomes was tested in vitro utilizing the dialysis bag diffusion method and compared with mucoadhesive in situ gel loaded with the plain drug suspension. The drug release profile from mucoadhesive in situ gel of the optimal GLIB-loaded bilosome formulation was subjected to release kinetics studies and evaluated for ‘goodness-of-fit’ into mathematical model equations, mainly zero order, first order, Higuchi matrix, Korsmeyer–Peppas, and Hixson–Crowell cube root equation. The releasing mechanism of the optimal GLIB-loaded bilosome formulation was comprehended by means of these kinetic models. The “best-fit” model for that formulation was determined to be the one whose R^2^ value was closest to 1.000, and this value was analyzed by employing an add-in program DDSolver [5,14].

### 2.12. In Vivo Brain Biodistribution Study of Optimal GLIB-Loaded Bilosomes

Swiss albino mice (female) weighing 30–40 g and aged 6–7 weeks were used for in vivo brain biodistribution study of the optimal formulation. Mice were kept at the Animal House Facility of Shoolini University of Biotechnology and Management Sciences (SUBMS) in Solan and were procured from the National Institute of Pharmaceutical Education and Research, Mohali. All animal procedures were approved by the SUBMS Institutional Animal Ethics Committee (protocol no. IAEC/SU/22/26), and the study was conducted as per guidelines set by the Committee for Control and Supervision of Experiments on Animals, Government of India. Twenty-four mice were divided into two groups. One group was given intranasal mucoadhesive in situ gel of GLIB-loaded bilosomes, and another group received mucoadhesive in situ gel of plain GLIB (0.39 mg/kg animal dose). For intranasal administration, mice were held in a supine position and formulation (5–10 µL) containing an equivalent dose of the drug was instilled into each nostril with the help of a micropipette. Three mice from each group were euthanized by cervical dislocation and mercifully sacrificed at intervals of 1 h, 4 h, 8 h, and 12 h. The brain sample was taken from each mouse and homogenized in 3 mL of acetonitrile/methanol (4:1 *v*/*v*) using a tissue homogenizer for 1 min at 24,000 rpm. To perform an HPLC analysis, brain samples were kept at −20 °C, and a sensitive and verified chromatographic condition was used. A reverse-phase column (C18, 50×2.1 mm) was utilized. The mobile phase comprised a water and acetonitrile mixture in the ratio of 30:70 (*v*/*v*). Isocratic chromatographic separation was performed at 40 °C and a flow rate of 0.8 mL/min. The mean concentration of the drug in the homogenized brain was plotted versus time [17,32].

### 2.13. Statistical Analysis

All values were expressed either as mean ± standard error of mean (SEM) or mean ± standard deviation (SD), as mentioned in the figure legends. Statistical analysis was performed using an unpaired Student *t*-test (parametric and two-tailed) to compare the two groups. The statistical analysis was performed using GraphPad Prism 10.4.0 software. *p* values less than 0.05 were considered statistically significant.

## 3. Results

### 3.1. FTIR Study

FTIR spectrums of GLIB, excipients, and the GLIB + excipient mixture were collected in transmittance mode. The FTIR spectrum for pure GLIB showed sharp characteristic peaks at frequencies 3322 cm_−1_ (N-H group), 1722 cm_−1_ (C-OH group), 2932.18 cm_−1_ (C-H group), and 1164 cm_−1_ (S=O group). The presence of all the major peaks of the drug in the drug–excipient mixture spectrum indicated the absence of any chemical interaction between the drug and excipients, which demonstrated no significant structural perturbation and confirmed their compatibility (reported in Supplementary Materials).

### 3.2. Preparation and Optimization of GLIB-Loaded Bilosomes

To investigate the effect of formulation-independent variables on the properties of the nanovesicles and ultimately recommend a formulation with an optimal composition, a total 17 bilosomal formulations were prepared based on Box–Behnken (3^2^) design (Table 2). The formulations were then subjected to characterization to determine the entrapment efficiency and in vitro drug release. Being close to 1.000, the values of R^2^ for the quadratic model implied an excellent fit of response surface polynomials to the response variable data. The model F-values for dependent variables Y_1_ and Y_2_ were found to be significant, i.e., *p* < 0.05. Lack of fit values for both the responses were found to be insignificant (*p* > 0.05), indicating the validity of the selected models. The closeness of the adjusted R^2^ (0.9588, 0.9728) and predicted R^2^ (0.7708, 0.8570) to actual the model R^2^ (0.9820, 0.9881) for both the responses also indicated the goodness of fit to the data. The predicted R^2^ value for the responses, i.e., entrapment efficiency and in vitro drug release, were in reasonable agreement with the adjusted R^2^, suggesting that the difference was less than 0.2 and implying that the model can be employed to navigate the design space. For both responses, the values for adequate precision were also found to be 16.067 and 24.235, which were satisfactorily greater than the required value of 4.0, implying the precision of the results. In addition, it can be concluded that the statistical model proposed in this study is significant (Table 3) [5,17,28,33,34].

The polynomial equation’s fitting of the terms suggested that the model was significant and would successfully traverse the design space [5,35]. The final polynomial equations for each response variable in terms of actual factors are given below:

Response 1 (% entrapment efficiency) Y_1_ = 64.96 − 3.37 × X_1_ − 4.87 × X_2_ + 0.5763 × X_3_ + 2.83 × X_1_X_2_ − 0.9225 × X_1_X_3_ − 4.32 × X_2_X_3_ − 15.58 × X_1_^2^ − 6.39 × X_2_^2^ − 10.28 × X_3_^2^ (4)

Response 2 (in vitro release efficiency at 12 h) Y_2_ = 87.91 − 5.45 × X_1_ + 1.47 × X_2_ + 0.1013 × X_3_ + 0.6025 × X_1_X_2_ + 0.56 × X_1_X_3_ − 1.16 × X_2_X_3_ + 2 × X_1_^2^ + 2.30 × X_2_^2^ + 1.83 × X_3_^2^ (5)

**Figure 1:**
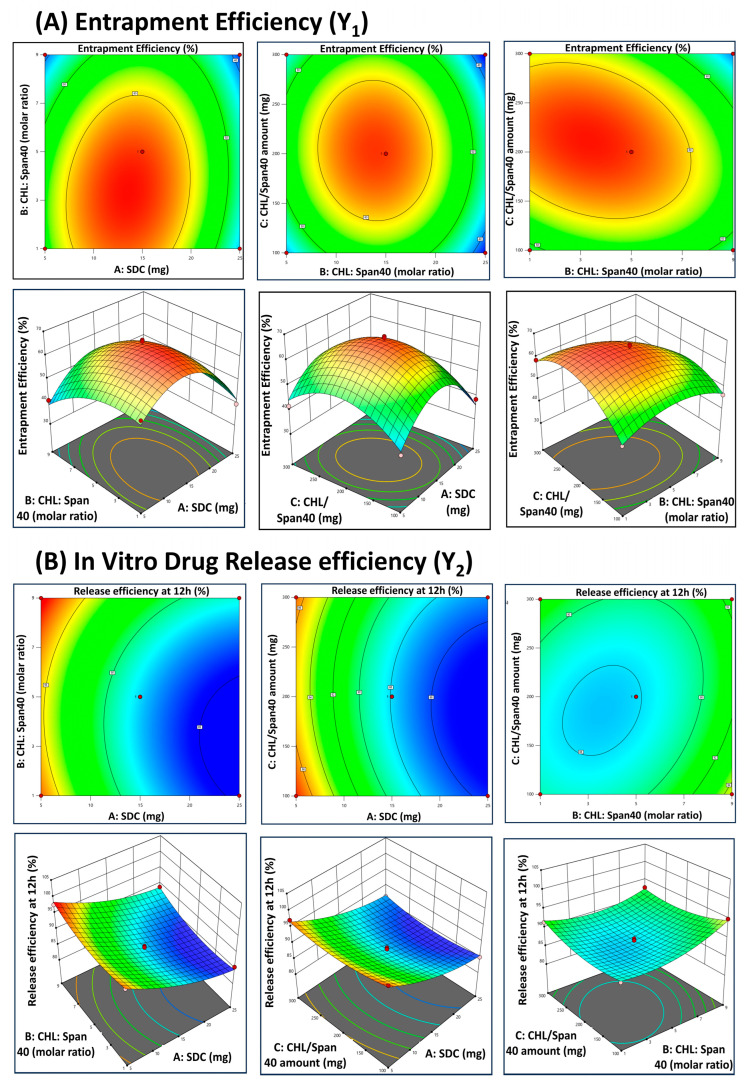
Two-dimensional contour and corresponding three-dimensional plots for depicting the effect of factors, i.e., amount of sodium deoxycholate (SDC) (X_1_), molar ratio of cholesterol (CHL):Span 40 (X_2_) and amount of CHL/Span 40 mixture (X_3_) on response (**A**) % entrapment efficiency (Y_1_) and (**B**) % in vitro drug release efficiency at 12 h (Y_2_) of GLIB-loaded bilosomes.

The two response surfaces, namely the percent entrapment efficiency and in vitro drug release efficiency against the factor levels of SDC (amount), CHL:Span 40 (molar ratio), and CHL/Span 40 (amount), in the analyzed models are depicted in contour and 3D plots in Figure 1A,B.

### 3.3. In Vitro Evaluation of Developed Formulation

#### 3.3.1. Entrapment Efficiency (EE%)

##### Effect of SDC (X_1_) on EE% (Y_1_)

The EE% of GLIB-loaded bilosomes is presented in Table 2, ranging from 35.76% to 66.76%. An increase in the amount of SDC leads to an increase in entrapment efficiency to a certain point and then a concomitant decrease in entrapment efficiency (Figure 1A). This trend is exemplified by formulations F3, F14, F15, and F16 with a minimum concentration of SDC (X_1_ = 5 mg), which showed the lowest entrapment efficiency (Y_1_) in the range of 39.56% to 55.87%. Formulations with the highest amounts of SDC (X_1_ = 25 mg) in F7, F8, F11, and F13 showed entrapment efficiency of 35.76% to 39.66%. Conversely, formulations with the optimum amount of SDC, i.e., 15 mg, in F1, F2, F4, F5, F6, F9, F10, F12, and F17 showed the highest entrapment efficiency in the range of 40.08% to 66.76% (Table 2). This effect could be imparted due to the hydrophobicity of SDC, which integrates the lipophilic drug into the hydrophobic part of the phospholipid bilayer of bilosomes, thereby increasing the EE%. The increased hydrophobicity of SDC also acted as a barrier, preventing drug leakage from the nanovesicles, leading to high entrapment efficiency [33,36]. A very high amount of bile salt causes a decrease in entrapment, which may be due to reduced vesicle compactness, and at higher concentration it will function as a solubilizing surfactant, which facilitates drug leakage and lowers the EE% [37]. ANOVA testing confirmed a significant effect of bile salt (X_1_) on the EE% (Y_1_) of bilosomes (*p* = 0.0054).

##### Effect of CHL:Span 40 Molar Ratio (X_2_) on EE% (Y_1_)

Formulations with low contents of Span 40 with respect to CHL in a molar mixture (F2, F6, F13, and F16) showed low EE%. For instance, formulations F4, F5, F9, F10, and F17 (repeat batches) with 1:5 molar ratios of CHL:Span 40 (X_2_) exhibited Y_1_ values in the highest range of 63.03% to 66.76%, respectively. Higher EE% is due to the presence of CHL, which provides rigidity and stability to the phospholipid bilayer with a subsequent decrease in drug leakage from nanovesicles [19,33]. Conversely, formulations with a higher CHL:Span 40 molar ratio (X_2_ = 1:9), i.e., F1, F11, F12, and F14, tend to exhibit lower entrapment efficiencies (Y_1_ in the range of 35.76% to 46.56%) due to the increased concentration of surfactant Span 40 as compared to CHL (Table 2). A higher concentration of surfactant as compared to CHL will possibly enhance drug partitioning in the dispersion medium due to micelle formation and fluidize the lipid bilayer, which will decrease the EE% [14,36].

##### Effect of CHL/Span 40 Amount (X_3_) on EE% (Y_1_)

Formulations with 200 mg of CHL/Span 40 (X_3_) generally exhibited higher entrapment efficiencies. Notably, formulations F4, F5, F9, F10, and F17, all containing 200 mg of X_3_, demonstrated higher Y_1_ values, as shown in Table 2. It is evident that adding a higher amount of CHL/Span 40 mixture in the formulation leads to significant increase in the EE% of the developed bilosomes, as depicted in Figure 1A. This observation may be explained by the fact that the CHL/Span 40 mixture serves as the structural scaffolding and primary material that forms bilosome vesicles. Consequently, elevated concentrations of the same may result in the formation of a greater number of these large vesicles, which, in turn, may accommodate greater concentrations of drug. Additionally, as less Span 40 is insufficient to stabilize the bilosomal membrane, lowering the Span 40 level in CHL/Span 40 mixture may result in greater drug leakage, thereby reducing drug entrapment [38,39].

#### 3.3.2. In Vitro Drug Release Study

**Figure 2:**
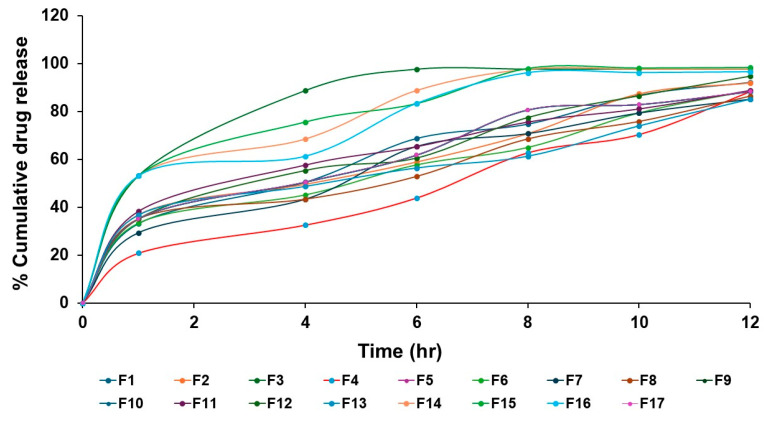
In vitro drug release profiles from GLIB-loaded bilosomal formulations (F1–F17) for 12 h in dissolution media containing PBS (pH 7.4) and ethanol (80:20).

The percent cumulative drug release at 12 h in PBS with ethanol (80:20) from the developed bilosomal formulation was found to be in the range of 85.13% to 98.08% for all the formulations, as shown in Figure 2. Increasing the amount of CHL/Span 40 mixture caused a significant decrease in drug release as the formed particles were bigger in size, leading to the formation of a viscous/thicker formulation, which, unfortunately, released the drug at a slower rate (Figure 1B). Figure 2 implies that the GLIB release from the developed bilosomal formulations was biphasic. An initial burst release of the drug was noticed within the first 1 h (20.79% to 53.03%), followed by a slower and sustained drug release (85.13% to 98.08%). The initial burst phase may be due to the adsorbed or surface drug in the release medium, while the later phase of drug release may be due to the removal of the drug adsorbed from within the vesicle. The steady partitioning of GLIB entrapped within the bilosome bilayer to the release medium causes the extended release. Conclusively, the dissolution behavior, characterized by a biphasic pattern, underscores the ability of the bilosomal formulation to provide both an initial therapeutic effect and a prolonged release phase. Notably, higher SDC concentrations tended to decrease both entrapment and release efficiencies, potentially due to the destabilization of the nanoparticulate system [40]. Also, the data from in vitro release for all the seventeen formulations were fitted into release kinetics models, which showed that the formulation followed the Higuchi model (shown in Supplementary Data). The Higuchi model characterizes drug release as predominantly diffusion-driven, with the release rate progressively declining over time in controlled intrusion systems. Over time, however, the drug’s release is significantly enhanced due to increased permeability within the polymer matrix as erosion advances [41].

### 3.4. Determination of Optimal Formulation

After considering the pattern and possible mechanism of drug release from different GLIB-loaded bilosomes, the next step was selection of the final optimal formulation. Numerical and graphical optimization techniques were employed to search for the optimal formulation within the experimental domain studied. The constraints were set to the desired goals, and the entire experimental domain was explored for the composition of the optimal solution where the set constraints were met within the range selected. The resulting optimized solution, with subsequent responses and desirability values, is given in Table 4.

**Figure 3:**
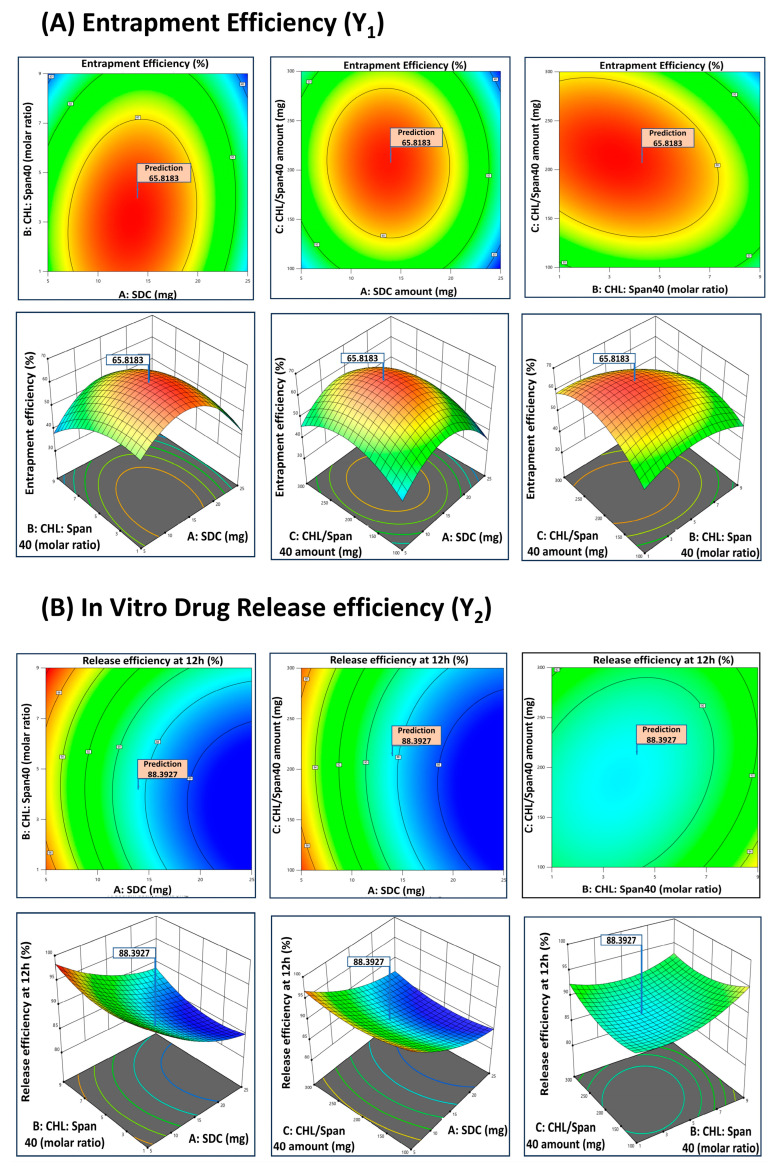
Two- and three-dimensional response surface plots to determine optimized formulation by numeric optimization technique for response (**A**) % entrapment efficiency (Y_1_) and (**B**) in vitro drug release efficiency at 12 h (Y_2_).

**Figure 4:**
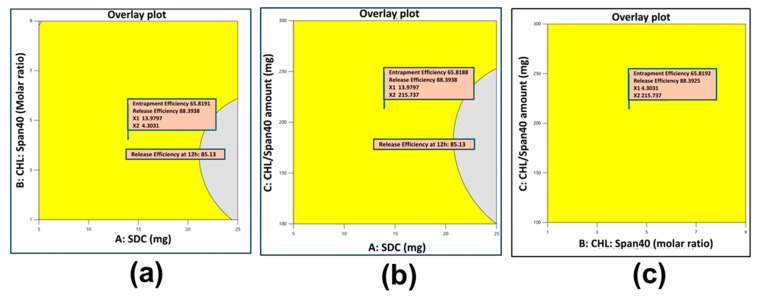
Overlay contour plots to determine optimized GLIB-loaded bilosomal formulation. (**a**) SDC amount vs. CHL:Span 40 molar ratio, (**b**) SDC amount vs. CHL/Span40 amount, and (**c**) CHL:Span 40 molar ratio vs.CHL/Span40 amount.

The results for numerical optimization are shown in Figure 3, and graphical optimization is shown in overlay plots in Figure 4. Results obtained by numerical and graphical optimization are confirmed by the 3D and overlay plots (Figure 3 and Figure 4) that showed the exact location of the optimal solution in the experimental domain. The optimal formulation suggested by design was prepared and coded as BF1. The optimal formulation was loaded into the mucoadhesive in situ gel and further evaluated for in vitro and in vivo studies.

### 3.5. Evaluation of Optimized GLIB-Loaded-Bilosomal Formulation

#### 3.5.1. Particle Size, Polydispersity Index (PDI), and Zeta Potential

**Figure 5:**
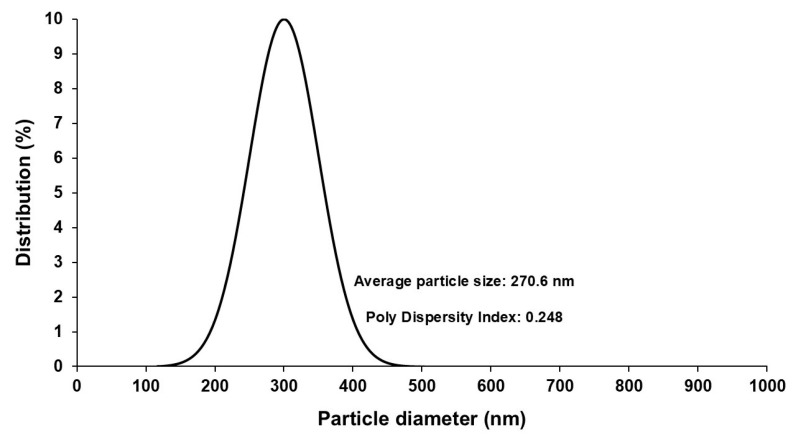
Particle size distribution of the optimized GLIB-loaded bilosomal formulation (BF1) as determined by zetasizer.

Particle size, PDI, and zeta potential of the optimal GLIB-loaded bilosome formulation (BF1) were determined by zetasizer. The particle size for BF1 was found to be 270.6 nm (Figure 5), indicating the formation of bilosomes with a small vesicle size and complying with the range to cross the olfactory region in the nasal cavity and reach the brain [42]. The PDI was 0.248, which suggested highly polydisperse vesicles, and the zeta potential was −37.3 mV, confirming sufficient negative charge on the formed bilosomes to ensure electric repulsion between them to prevent their aggregation. The negative charge is due to SDC, an anionic bile salt, present in the formulation.

#### 3.5.2. Transmission Electron Microscopy (TEM)

**Figure 6:**
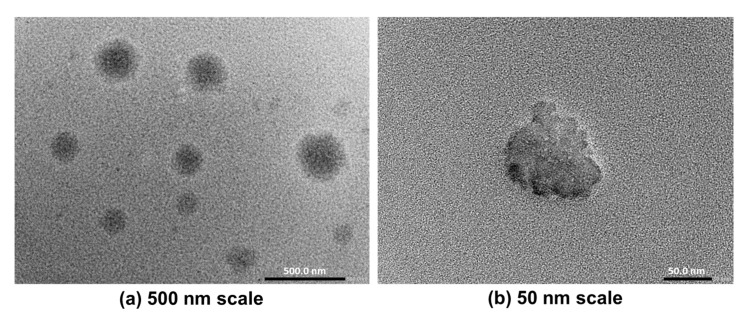
Transmission electron micrograph of the optimized GLIB-loaded bilosomal formulation (BF1) at a scale of 500 nm (**a**) and 50 nm (**b**).

The TEM microscopy images of the optimized GLIB-loaded bilosomal formulation (BF1) illustrated non-aggregating unilamellar nanovesicles, which are spherical in shape. These vesicles exhibited reduced propensity for agglomeration, which is advantageous for ensuring the stability of nanoparticles (Figure 6).

#### 3.5.3. Differential Scanning Electron (DSC) Study

**Figure 7:**
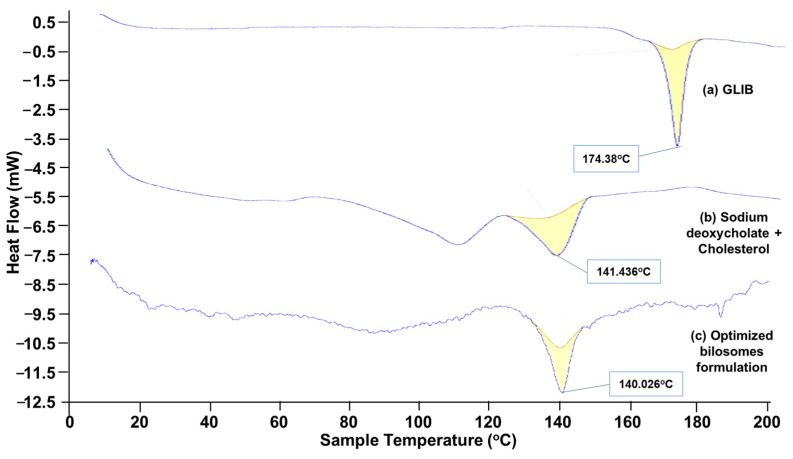
DSC thermogram of (**a**) glibenclamide (GLIB), (**b**) the excipients (SDC and CHL), and (**c**) the optimized bilosomal formulation.

The DSC thermograms of GLIB, the physical mixture of excipients (CHL and SDC), and the optimal formulation (BF1) are shown in Figure 7. Figure 7a clearly shows the DSC thermogram of GLIB with a sharp endothermic peak at 174.384 °C corresponding to its melting point [43]. The overlay DSC thermogram for a physical mixture of excipients and optimized formulation has shown endothermic peaks at 141.43 °C and 140.02 °C, respectively, in Figure 7b,c. The DSC curve for GLIB-loaded bilosome formulation BF1 exhibited no endothermic peak of pure drug, which might be due to its encapsulation in the nanovesicles. When the drug is encapsulated in a bilosome nanovesicle, it may not be in its pure crystalline form. The drug may be solubilized or dispersed at the molecular level within the vesicle’s matrix. The drug may not show its usual melting tendency in this state, which is typically seen when the drug is in a crystalline form [14,44]. Notably, the DSC analysis revealed no discernible evidence of molecular interactions between GLIB and the employed excipients for the preparation of bilosomes.

#### 3.5.4. Entrapment Efficiency (EE%) of Optimal Formulation

The EE% of the optimal GLIB-loaded bilosomal formulation (BF1) was found to be 68.39%. The highest drug entrapment in BF1 could be due to enhanced stability, fluidity of lipid membrane in nanovesicles, and increased drug solubility due to the presence of bile salt [14] and surfactant, as discussed previously, under the effects of formulation variables on EE%.

#### 3.5.5. In Vitro Drug Release Study of Optimal Formulation

**Figure 8:**
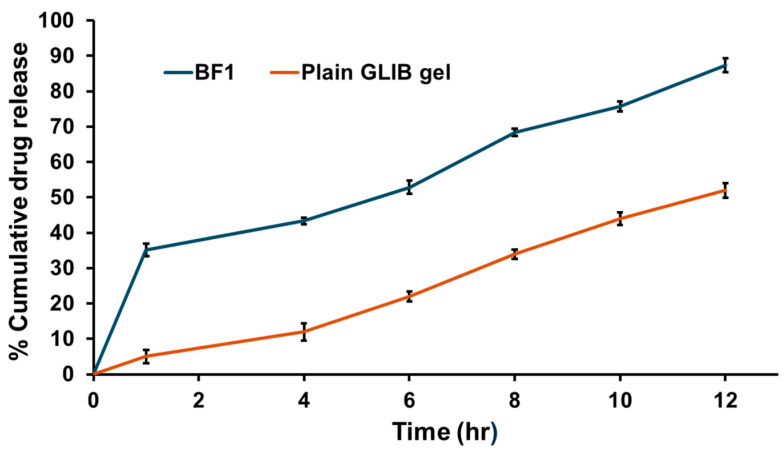
In vitro drug release profiles from the mucoadhesive in situ gel of optimal GLIB-loaded bilosome formulation (BF1) and mucoadhesive in situ gel of plain GLIB at 12 h. Data are represented as mean ± SD, *n* = 3.

In vitro drug release of GLIB from mucoadhesive in situ gel of optimal GLIB-loaded bilosomal formulation (BF1) at 12 h was found to be 87.29 ± 1.98% as compared to the 52.01 ± 2.04% from mucoadhesive in situ gel loaded with GLIB suspension, as shown in Figure 8. These results confirm that developed bilosomal formulation BF1 showed a gradual sustained drug release for 12 h, which may be attributed to the ability of bilosomes to act as a drug reservoir and penetrate easily into the nasal mucosa. Enhanced drug solubility was majorly due to the incorporation of bile salts and nanosized vesicles, which resulted in a nano-solubilization effect [33]. Furthermore, a biphasic pattern with a rapid initial burst release in the first 1 h (35.12 ± 1.74%) followed by a slower release phase was noticed in GLIB-loaded bilosomes, which might be due to the detachment of drug adsorbed on the nanovesicles’ outer surface. Later, the extended drug release phase is attributed to the steady partitioning of the drug entrapped within the vesicular bilayer to the release medium. To add to it, bilosomes loaded into mucoadhesive polymer and in situ gel formulation prolonged the residence time of formulation in the nasal cavity, gradually enhancing the penetration of nanovesicles through olfactory mucosa and thereby enhancing the concentration of drug in the brain. The observed trend in drug release in optimal formulation BF1 suggested that lower concentrations of SDC, coupled with a moderate molar ratio and higher amounts of CHL/Span 40, enhanced the drug entrapment while maintaining a desirable drug release profile. On the other hand, drug release from the plain GLIB was poor, presumably due to its poor aqueous solubility. Furthermore, as per release kinetics modelling, Higuchi was the best-fit kinetic model with an R^2^ value of 0.9807 for the drug release data of BF1, showing diffusion-controlled drug release. This model states that the concentration of drug release increases as a function of the square root of time [45].

To validate the optimization process, we compared the predicted and experimental response variables Y_1_ (entrapment efficiency) and Y_2_ (in vitro release efficiency at 12 h) of the optimal formulation BF1, as shown in Table 5. The small percent deviation as an absolute value between the observed and predicted results proves the validation of the selected model [38]. The experimental results exhibited minimal deviation from the theoretical calculations, as evidenced by prediction errors consistently below 6% for each measured response [46].

#### 3.5.6. Sol-to-Gel Transition Temperature and Time

The sol-to-gel transition temperature of the developed mucoadhesive in situ gel was found to be 33.84 ± 0.29 °C, which is in accordance with the suitable range for intranasal administration. The sol-to-gel transition time was found to be 13.00 ± 0.85 s [47].

#### 3.5.7. Rheological Evaluation of Mucoadhesive In Situ Gel of Optimal GLIB-Loaded Bilosomes

**Figure 9:**
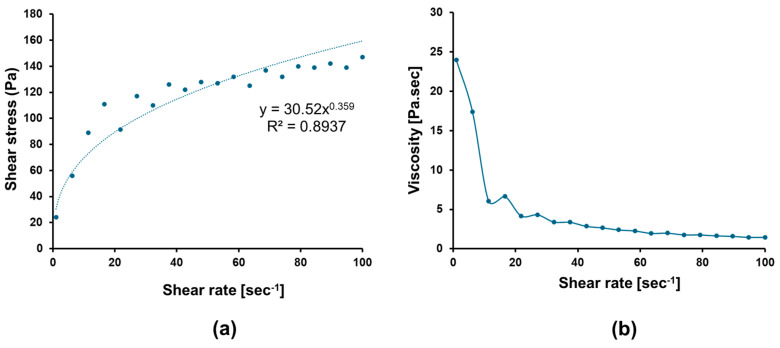
Rheogram of optimized mucoadhesive in situ gel formulation of GLIB-loaded bilosomes (BF1) showing (**a**) plot of shear stress (Pa) vs. shear rate (sec^−1^) and (**b**) the plot of viscosity (Pa.sec) vs. shear rate.

From the shear stress vs. shear rate plot (Figure 9a), the power law equation and trend line are calculated and used to determine rheological properties, such as the consistency (k) and flow index (n) of the gel formulation. The consistency index for in situ gel of BF1 was found to be 30.52 Pa.s^n^ and the flow index was 0.359. The R^2^ value for the curve fitting line was 0.89, suggesting goodness of fit for power law. The flow index value of 0.359 implied the shear thinning or pseudoplastic behavior of the gel formulation (n < 1). Moreover, as the shear rate increased from 1 to 100 sec^−1^, the viscosity readings ranged from 24 to 1.47 Pa.s, indicating a decrease in viscosity, as shown in Figure 9b. The average viscosity was found to be 4.67 Pa.s. It was concluded from the results that the gel formulation had sufficient consistency and good flowability for intranasal administration [17,35].

At low shear rates of BF1-loaded in situ gel, the viscosity increased, improving adhesion in the nasal canal and reducing the tendency to flow out. Therefore, BF1 dispersed in poloxamer 407, with HPMC K4M functioning as a mucoadhesive agent, could improve GLIB’s local residence period and regulate its release. Similar findings were made by Cunha et al. (2021), concluding that a favorable rheology can ensure that in situ intranasal formulations enter these areas easily and change into stationary gel, which results in a longer retention period and improved delivery of the drug [48].

The gel’s pH was found to be 6.1, which complies with the optimal pH range (5.7–6.3) for delivery systems suitable for intranasal administration. As a result, the developed formulations can be applied to the nose without causing interference with the nasal mucosa [49].

#### 3.5.8. In Vivo Brain Biodistribution Study

**Figure 10:**
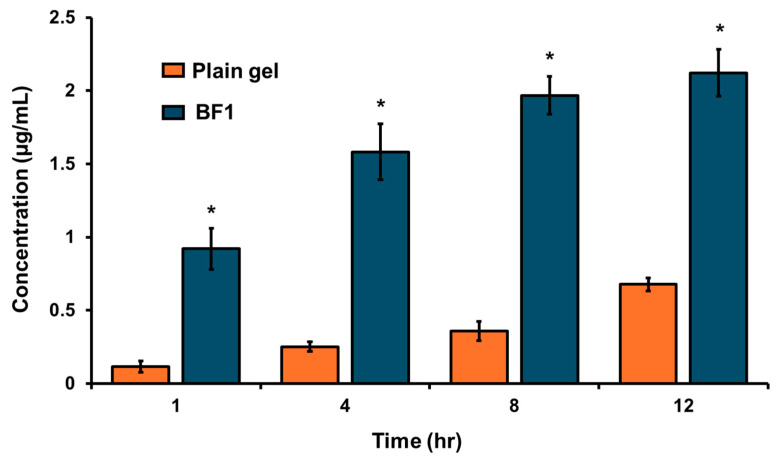
In vivo brain biodistribution study. GLIB concentration in the brain of Swiss albino mice after intranasal administration of mucoadhesive in situ gel of GLIB-loaded bilosomal formulation (BF1) and mucoadhesive in situ gel of plain GLIB at different time points for up to 12 h. GLIB concentration was found to be significantly increased in BF1 as compared to plain gel at all time points. Statistical analysis was performed using an unpaired Student *t*-test (parametric and two-tailed) to compare BF1 and plain gel. Data are represented as mean ± SEM; * *p* < 0.05 (*n* = 3).

The in vivo brain biodistribution study in Swiss albino mice demonstrated that intranasal administration of a mucoadhesive in situ gel containing GLIB-loaded bilosomes (BF1) resulted in significantly higher drug concentrations in the brain compared to the plain in situ gel, starting from 1 h onward. The brain drug concentrations for BF1 at 1, 4, 8, and 12 h were 0.92 ± 0.11, 1.58 ± 0.19, 1.96 ± 0.13, and 2.12 ± 0.16 µg/mL, respectively. In contrast, the plain in situ gel of plain drug suspension showed markedly lower concentrations of 0.12 ± 0.04, 0.25 ± 0.03, 0.36 ± 0.06, and 0.68 ± 0.04 µg/mL at the same points (Figure 10). These findings indicate that BF1 is not only a more effective formulation for achieving higher brain penetration but also enables faster drug delivery to the brain compared to the plain drug suspension. Although the drug’s poor solubility in its suspension form led to inadequate absorption despite intranasal delivery, BF1 demonstrated enhanced brain penetration of GLIB. This improvement may be attributed to increased drug dissolution when incorporated into bilosomes, as well as the enhanced penetration of nanovesicles through the nasal olfactory mucosa, facilitated by the presence of bile salts. To add to this, the small particle size of the vesicles, along with their flexibility and deformability, is also a plausible reason for enhanced extracellular and intracellular GLIB transport through olfactory mucosa directly to the brain [17,50].

## 4. Discussion

The results of this study provided crucial insights into the formulation, entrapment efficiency (EE%), in vitro drug release characteristics, and in vivo brain distribution of GLIB-loaded bilosomes. GLIB’s structural integrity in the mixture is crucial for preserving its therapeutic efficacy, and the FTIR analysis verified that there was no chemical interaction between GLIB and its excipients. The effects of formulation variables, including the amount of CHL, SDC, and Span 40, on bilosomal functions were investigated using a Box–Behnken design. A quadratic model was used to interpret the results of an evaluation of 17 bilosomal formulations for entrapment efficiency and in vitro drug release. The high R^2^ values for both responses (EE% and drug release) showed that the model had excellent predictive capability. The model’s robustness was further validated by the proximity of adjusted R^2^ and anticipated R^2^, which showed that the formulation variables had a substantial impact on the results. The concentration of SDC has a significant impact on entrapment efficiency [33]. The findings showed that the noptimum SDC concentration (15 mg) increased GLIB’s entrapment efficiency by up to 66.76% (Figure 1, Table 2). Formulations showed less entrapment at lower SDC concentrations (5 mg), but EE% dropped at higher concentrations (25 mg) as a result of bilosome instability. In line with earlier findings on the behavior of bile salt surfactants, this drop can be attributed to the solubilizing activity of excess bile salts, which causes vesicle fluidity and drug leakage. Additionally, CHL was essential for preserving the stability of the bilosomes. Because CHL gave the formulations their stiffness, those with an ideal CHL:Span 40 molar ratio of 1:5 (such as F4, F5, F9, F10, and F17) had greater EE% (Table 2).

In in vitro drug release studies, biphasic drug release, with an initial burst release followed by a sustained release, was observed from the developed GLIB-loaded bilosomes. The surface-adsorbed GLIB is responsible for this burst release, which reached up to 53.03% in the first hour. Slow partitioning of the drug from the bilosomal bilayer is probably what caused the subsequent persistent release, which reached up to 98.08% over 12 h (Figure 2). The investigation verified that longer drug release was caused by thicker formulations and larger particles at higher CHL and Span 40 concentrations. The structural function of cholesterol, which affects drug release kinetics in addition to stabilizing the bilosomal membrane, is consistent with this observation. However, the entrapment and release efficiency were reduced by greater SDC concentrations due to system destabilization.

In order to determine the optimal formulation based on the intended entrapment efficiency and drug release profile, the study used both numerical and graphical optimization techniques. By balancing the levels of SDC, CHL, and Span 40 within the experimental domain, the ideal formulation was found, resulting in a formulation with controlled drug release and good entrapment efficiency. The optimal formulation was loaded into mucoadhesive in situ gel formulation to enhance the drug retention time in the nasal cavity. The optimized GLIB-loaded bilosomal formulation (BF1) has been evaluated further for particle size, PDI, zeta potential, surface morphology, DSC, EE%, rheology, pH, and in vitro drug release. In contrast to the mucoadhesive in situ gel loaded with GLIB suspension, which showed a 52.01% release at 12 h, the GLIB-loaded bilosomes showed a sustained release pattern with an 87.29% release at 12 h (Figure 8). This notable variation demonstrates the advantages of the bilosomal formulation, where the drug is released gradually in a controlled manner because of the reservoir function of the nanovesicles. Furthermore, in the in vivo brain biodistribution study, the improved effectiveness of the optimal GLIB-loaded bilosomal formulation (BF1) in comparison to the plain GLIB suspension is directly demonstrated. In comparison to the in situ gel loaded with GLIB suspension (0.68 ± 0.04 µg/mL), the brain concentration of GLIB from BF1 (2.12 ± 0.16 µg/mL) was considerably greater after 12 h, indicating the superiority of the bilosomal system in improving brain penetration (Figure 10).

Overall, the noticeably increased levels of GLIB in the brain after BF1 treatment indicated that the bilosomal formulation not only increases drug solubility but also improves brain targeting via the nasal route. Because of this, BF1 is a very promising formulation for drug transport to the central nervous system (CNS), especially for medications like GLIB that have limited solubility. In conclusion, the developed formulation offers a promising intranasal substitute over plain drug suspension with boosted therapeutic efficacy for treating brain diseases. Bile salts present in the bilosomes act as an edge activator and enhance the stability and effectiveness of the nanovesicles, mainly for crossing the BBB to deliver the neuroprotective agents to the brain. Thus, bilosomes appear promising in their ability to deliver therapeutic substances and can be further explored for their clinical applications in treating a variety of neurological disorders.

Moving forward, the future scope of this study includes exploring the therapeutic effects of the GLIB-loaded bilosomal formulation in neurological disorders, such as Alzheimer’s, stroke, and Parkinson’s disease, preclinically. Furthermore, optimization and scale-up of the formulation could facilitate its clinical applications. Ultimately, advancing this technology could significantly improve the treatment outcomes for patients with neurological disorders, particularly those that are difficult to treat via traditional oral drug delivery methods.

## Figures and Tables

**Table 1:** Design specifications and test setups for the three-factor, two-level (3^2^) Box–Behnken design.

Independent Variables (Factors)	Levels	
	Low (−1)	High (+1)
Amount of SDC (mg): X_1_	5	25
CHL:Span 40 molar ratio (*w*/*w*): X_2_	1:1	1:9
Amount of CHL/Span 40 mixture (mg): X_3_	100	300
**Dependent variables (Responses)**		**Desirability constraints**
Entrapment efficiency (%) Y_1_		In range (40–70%)
In vitro release efficiency (%) Y_2_		In range (80–100%)

**Table 2:** Experimental runs, independent variables, and measured responses of Box–Behnken (3^2^) design for the prepared GLIB-loaded bilosomes.

Formulations	Amount of SDC (mg) (X_1_)	CHL:Span 40 (Molar Ratio) (X_2_)	CHL/Span 40 Amount (mg) (X_3_)	Entrapment Efficiency (%) (Y_1_)	In Vitro Release Efficiency (%) (Y_2_)
F1	15	1:9	300	40.08	92.79
F2	15	1:1	300	58.65	91.43
F3	5	1:5	300	41.56	97.22
F4	15	1:5	200	64.69	87.05
F5	15	1:5	200	66.19	87.95
F6	15	1:1	100	47.87	88.97
F7	25	1:5	100	38.47	85.13
F8	25	1:5	300	36.78	86.51
F9	15	1:5	200	66.76	88.45
F10	15	1:5	200	64.14	88.02
F11	25	1:9	200	35.76	88.93
F12	15	1:9	100	46.56	94.96
F13	25	1:1	200	39.66	85.51
F14	5	1:9	200	40.67	97.69
F15	5	1:5	100	39.56	98.08
F16	5	1:1	200	55.87	96.68
F17	15	1:5	200	63.03	88.07

Note: GLIB concentration was kept constant (3 mg) for all formulation.

**Table 3:** Statistical parameters for response variables Y_1_ and Y_2_ with respect to factor terms X_1_, X_2_, and X_3_ obtained using ANOVA and multiple linear regression analysis.

Parameters	Response *Y*_1_ (Entrapment Efficiency)	Response *Y*_2_ (In Vitro Release Efficiency at 12 h)
Model (Quadratic)	Significant (*p* < 0.0001)	Significant (*p* < 0.0001)
Model term X_1_	*p* = 0.0054	*p* < 0.0001
Model term X_2_	*p* = 0.0007	*p* = 0.0003
Model term X_3_	*p* = 0.5191	*p* = 0.6650
Model term X_1_^2^	*p* < 0.0001	*p* = 0.0008
Model term X_2_^2^	*p* = 0.0009	*p* = 0.0003
Model term X_3_^2^	*p* < 0.0001	*p* = 0.0015
Model term X_1_X_2_	*p* = 0.0509	*p* = 0.0990
Model term X_1_X_3_	*p* = 0.4674	*p* = 0.1205
Model term X_2_X_3_	*p* = 0.0088	*p* = 0.0081 (*p* < 0.05)
Lack of fit	Non-significant (*p* > 0.05)	Non-significant (*p* > 0.05)
R²	0.9820	0.9881
Adjusted R²	0.9588	0.9728
Predicted R²	0.7708	0.8570
Adeq. Precision	16.0671	24.2359
**Coefficients**		
A_0_ (Intercept)	64.96	87.91
A_1_ (X_1_) SDC	−3.37	−5.45
A_2_ (X_2_) CHL:Span 40	−4.87	1.47
A_3_ (X_3_) CHL/Span 40	0.5763	0.1013
A_4_ (X_1_^2^)	−15.58	2.00
A_5_ (X_2_^2^)	−6.39	2.30
A_6_ (X_3_^2^)	−10.28	1.83
A_7_ (X_1_X_2_)	2.83	0.6025
A_8_ (X_1_X_3_)	−0.9225	0.5600
A_9_ (X_2_X_3_)	−4.32	−1.16

**Table 4:** Determination of optimal formulation of GLIB-loaded bilosomes using the numeric optimization technique.

Solutions Obtained Using Numerical Optimization					
Amount of SDC	CHL:Span 40 (molar ratio)	CHL/Span 40 (amount)	% Entrapment efficiency	% In vitro release efficiency	Desirability
13.98 mg	1:4.3	215.737 mg	65.81%	88.39%	1.000

**Table 5:** Comparative predicted vs. experimental values of different response variables for validation of optimized formulation BF1.

S. No.	Response	Predicted Value	Experimental Value	% Prediction Error
1.	Entrapment efficiency (%)	65.818%	68.39%	3.76
2.	% in vitro release efficiency at 12 h	88.393%	87.29%	−1.26

## Data Availability

The original contributions presented in this study are included in the article. Further inquiries can be directed to the corresponding authors.

## Supplementary Materials

The following supporting information can be downloaded at https://www.mdpi.com/article/10.3390/pharmaceutics17020193/s1: Figure S1 FTIR spectrum of glibenclamide (GLIB), excipients [cholesterol (CHL), sodium deoxycholate (SDC), Span 40], and physical mixture of GLIB and excipients; Table S1: Mathematical model fitting values obtained from percent drug release profile of glibenclamide loaded bilosomes (F1–F17). Supplementary Data: Chromatograms for in vivo brain distribution data.
