# Supplementary material for: Intranasal Mucoadhesive In Situ Gel of Glibenclamide-Loaded Bilosomes for Enhanced Therapeutic Drug Delivery to the Brain
Source: Pharmaceutics. 2025 Feb 4;17(2):193. doi: 10.3390/pharmaceutics17020193 (PMC11859129; doi:10.3390/pharmaceutics17020193)
Supplement: Supplementary file 1 [file pharmaceutics-17-00193-s001.zip › Chromatograms for in vivo brain distribution data.pdf]

Chromatograms for *In vivo* Brain distribution

Plain gel 1 hr

Area % Report

Data File: D:\EZChrom Elite\Enterprise\Projects\Default\Data\SU-HK-MT-53.dat
Method: D:\EZChrom Elite\Enterprise\Projects\Default\Method\untitled.met
Acquired: 6/17/2023 1:56:20 PM
Printed: 6/17/2023 5:09:19 PM

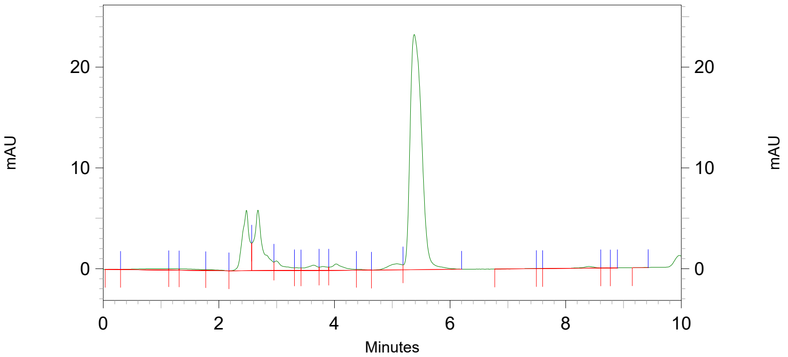

DAD: Signal D, 230 nm/Bw:4 nm Results

Retention Time	Area	Area %	Height	Height %
0.133	787	0.08	63	0.08
1.020	9861	0.96	293	0.35
1.153	3194	0.31	301	0.36
1.333	6737	0.65	290	0.35
1.820	2762	0.27	181	0.22
2.480	104997	10.18	12560	15.13
2.673	127713	12.38	12592	15.17
2.993	22627	2.19	2005	2.42
3.347	3743	0.36	577	0.70
3.640	14582	1.41	1094	1.32
3.800	7318	0.71	804	0.97
4.027	18870	1.83	1326	1.60
4.427	1673	0.16	198	0.24
5.080	21087	2.04	1270	1.53
5.380	678186	65.77	48946	58.98
7.413	1085	0.11	46	0.06
7.540	119	0.01	28	0.03
8.400	5314	0.52	339	0.41
8.720	248	0.02	32	0.04
8.840	82	0.01	21	0.03
9.380	207	0.02	28	0.03

Totals	1031192	100.00	82994	100.00

Plain gel 4 hr

Area % Report

Data File: D:\EZChrom Elite\Enterprise\Projects\Default\Data\SU-HK-MT-60.dat
Method: D:\EZChrom Elite\Enterprise\Projects\Default\Method\untitled.met
Acquired: 6/17/2023 3:50:15 PM
Printed: 6/17/2023 5:14:35 PM

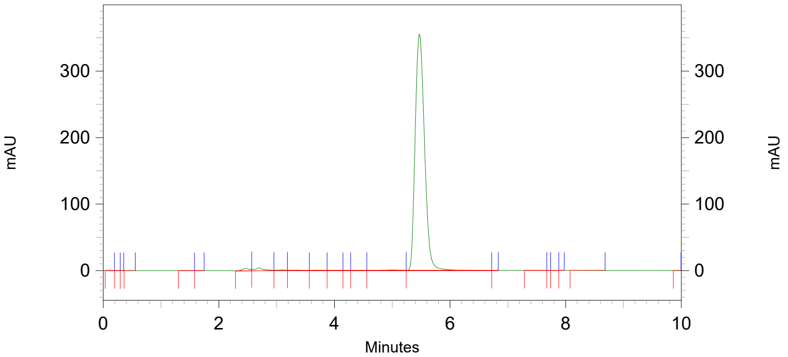

DAD: Signal D, 230 nm/Bw:4 nm Results

Retention Time	Area	Area %	Height	Height %
0.073	266	0.00	36	0.00
0.220	121	0.00	27	0.00
0.313	33	0.00	13	0.00
0.433	126	0.00	16	0.00
1.467	352	0.00	38	0.00
1.620	136	0.00	21	0.00
2.473	58335	0.67	7034	0.91
2.693	96463	1.11	8773	1.14
3.093	25991	0.30	2421	0.31
3.280	29425	0.34	1751	0.23
3.693	14438	0.17	1334	0.17
3.987	7791	0.09	612	0.08
4.187	2591	0.03	342	0.04
4.473	4566	0.05	282	0.04
5.007	40124	0.46	1749	0.23
5.467	8372717	96.65	744806	96.73
6.780	228	0.00	41	0.01
7.427	1306	0.02	100	0.01

7.700	63	0.00	22	0.00
7.773	124	0.00	23	0.00
7.920	43	0.00	12	0.00
8.400	7700	0.09	501	0.07
9.960	90	0.00	18	0.00
Totals	8663029	100.00	769972	100.00

Plain gel 8 hr

Area % Report

Data File: D:\EZChrom Elite\Enterprise\Projects\Default\Data\SU-HK-MT-54.dat
Method: D:\EZChrom Elite\Enterprise\Projects\Default\Method\untitled.met
Acquired: 6/17/2023 2:07:54 PM
Printed: 6/17/2023 5:10:31 PM

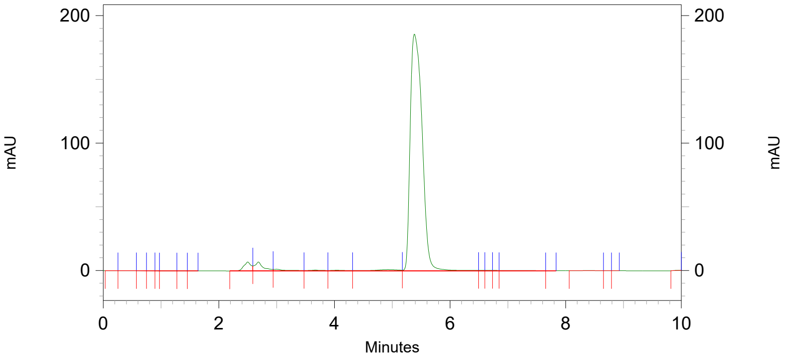

DAD: Signal D, 230 nm/Bw:4 nm Results

Retention Time	Area	Area %	Height	Height %
0.053	211	0.00	15	0.00
0.273	332	0.01	21	0.00
0.613	131	0.00	20	0.00
0.767	125	0.00	15	0.00
0.907	67	0.00	23	0.01
1.007	132	0.00	13	0.00
1.313	122	0.00	23	0.01
1.487	114	0.00	17	0.00
2.500	126133	2.11	14779	3.45
2.687	148708	2.48	14680	3.43
2.993	39548	0.66	2672	0.62
3.667	20272	0.34	1319	0.31
4.033	18261	0.30	1155	0.27

4.927	55094	0.92	1905	0.44
5.380	5552257	92.70	389131	90.87
6.533	3059	0.05	492	0.11
6.687	3625	0.06	455	0.11
6.773	2947	0.05	446	0.10
6.873	11443	0.19	418	0.10
7.700	298	0.00	48	0.01
8.413	4615	0.08	353	0.08
8.687	66	0.00	17	0.00
8.840	49	0.00	16	0.00
9.967	1564	0.03	206	0.05
Totals	5989173	100.00	428239	100.00

Plain gel 12 hr

Area % Report

Data File: D:\EZChrom Elite\Enterprise\Projects\Default\Data\SU-HK-MT-56.dat
Method: D:\EZChrom Elite\Enterprise\Projects\Default\Method\untitled.met
Acquired: 6/17/2023 2:36:58 PM
Printed: 6/17/2023 5:12:07 PM

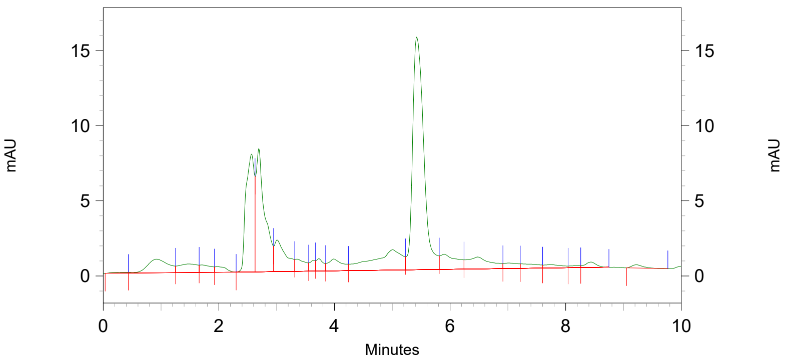

DAD: Signal D, 230 nm/Bw:4 nm Results

Retention Time	Area	Area %	Height	Height %
0.360	2333	0.18	120	0.13
0.913	48318	3.82	1908	2.07
1.480	26640	2.10	1202	1.30
1.713	14722	1.16	1045	1.13
1.973	10252	0.81	806	0.87
2.567	163911	12.94	16466	17.85
2.693	184199	14.55	17210	18.66

3.007	62101	4.90	4402	4.77
3.353	20742	1.64	1703	1.85
3.640	9893	0.78	1511	1.64
3.733	14617	1.15	1711	1.86
3.987	28621	2.26	1647	1.79
5.007	99377	7.85	2843	3.08
5.420	435878	34.42	32480	35.22
5.900	42405	3.35	2113	2.29
6.473	47173	3.73	1662	1.80
7.007	12895	1.02	775	0.84
7.240	12659	1.00	632	0.69
7.740	9896	0.78	468	0.51
8.213	3582	0.28	301	0.33
8.440	10043	0.79	758	0.82
9.227	6075	0.48	461	0.50
Totals	1266332	100.00	92224	100.00

BF1 1 hr

Area % Report

Data File: D:\EZChrom Elite\Enterprise\Projects\Default\Data\SU-HK-MT-57.dat
Method: D:\EZChrom Elite\Enterprise\Projects\Default\Method\untitled.met
Acquired: 6/17/2023 2:48:54 PM
Printed: 6/17/2023 5:12:53 PM

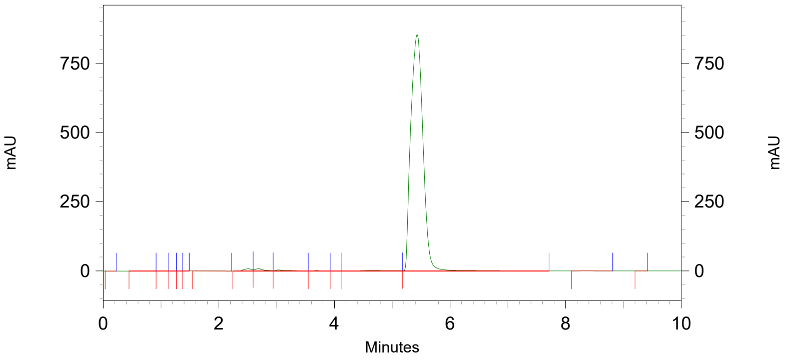

DAD: Signal D, 230 nm/Bw:4 nm Results

Retention Time	Area	Area %	Height	Height %
0.067	172	0.00	26	0.00
0.820	3836	0.01	200	0.01
0.947	1678	0.01	157	0.01

1.167	563	0.00	86	0.00
1.327	283	0.00	48	0.00
1.407	118	0.00	33	0.00
1.980	6368	0.02	332	0.02
2.507	160085	0.60	16969	0.92
2.687	194517	0.72	18683	1.01
3.033	126570	0.47	7819	0.42
3.693	31758	0.12	3306	0.18
4.020	5560	0.02	516	0.03
4.640	133319	0.50	3582	0.19
5.427	26205224	97.48	1789136	97.15
8.393	11493	0.04	752	0.04
9.340	196	0.00	28	0.00
Totals	26881740	100.00	1841673	100.00

BF1 4 hr

Area % Report

Data File: D:\EZChrom Elite\Enterprise\Projects\Default\Data\SU-HK-MT-55.dat
Method: D:\EZChrom Elite\Enterprise\Projects\Default\Method\untitled.met
Acquired: 6/17/2023 2:19:20 PM
Printed: 6/17/2023 5:11:06 PM

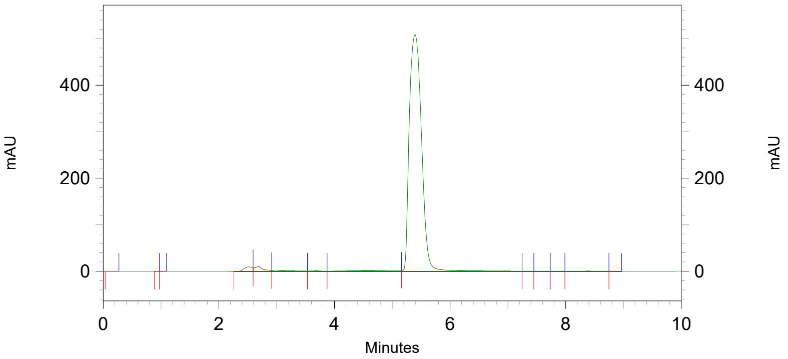

DAD: Signal D, 230 nm/Bw:4 nm Results

Retention Time	Area	Area %	Height	Height %
0.147	261	0.00	32	0.00
0.907	28	0.00	13	0.00
1.020	50	0.00	11	0.00
2.520	192638	1.17	20067	1.78
2.680	225129	1.37	22230	1.98

2.980	102974	0.63	5524	0.49
3.687	32709	0.20	2585	0.23
5.073	225802	1.37	4728	0.42
5.393	15639919	94.99	1066653	94.85
7.273	8523	0.05	737	0.07
7.467	10072	0.06	664	0.06
7.760	6858	0.04	501	0.04
8.400	18759	0.11	800	0.07
8.793	373	0.00	54	0.00
Totals	16464095	100.00	1124599	100.00

BF1 8 hr

Area % Report

Data File: D:\EZChrom Elite\Enterprise\Projects\Default\Data\SU-HK-MT-06.dat
Method: D:\EZChrom Elite\Enterprise\Projects\Default\Method\SU-DNK-VR.met
Acquired: 4/21/2023 12:44:56 PM
Printed: 4/26/2023 2:43:44 PM

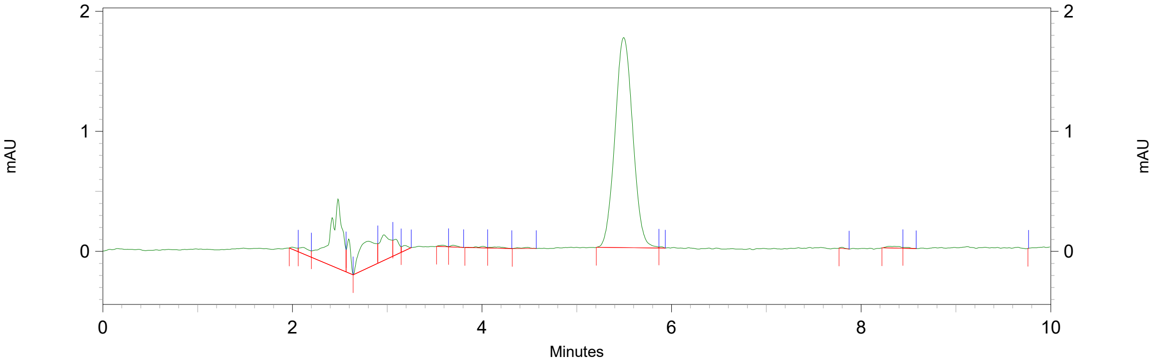

DAD: Signal D, 230 nm/Bw:4 nm Results

Retention Time	Area	Area %	Height	Height %
0.120	268	0.08	38	0.13
0.253	81	0.02	22	0.08
1.153	34	0.01	9	0.03
1.993	1121	0.34	198	0.70
2.100	590	0.18	150	0.53
2.480	17734	5.36	1997	7.05
2.593	4143	1.25	1249	4.41
2.767	7649	2.31	585	2.06
2.993	9142	2.76	1352	4.77
3.087	9911	2.99	1301	4.59
3.367	403	0.12	66	0.23

4.773	67	0.02	21	0.07
4.987	277	0.08	31	0.11
5.493	279579	84.44	21297	75.16
8.967	88	0.03	19	0.07
Totals	331087	100.00	28335	100.00

BF1 12 hr

Area % Report

Data File: D:\EZChrom Elite\Enterprise\Projects\Default\Data\SU-HK-MT-22.dat
Method: D:\EZChrom Elite\Enterprise\Projects\Default\Method\SU-DNK-VR.met
Acquired: 4/22/2023 12:40:52 PM
Printed: 4/26/2023 2:56:41 PM

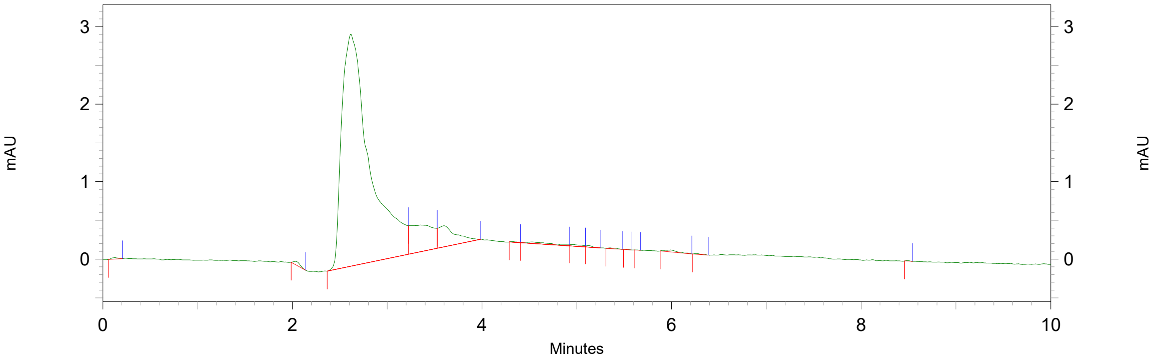

DAD: Signal D, 230 nm/Bw:4 nm Results

Retention Time	Area	Area %	Height	Height %
0.147	211	0.05	35	0.16
1.387	152	0.04	16	0.07
1.600	76	0.02	17	0.08
2.027	8035	1.99	996	4.57
2.613	301043	74.46	13350	61.21
3.427	48236	11.93	2535	11.62
3.613	23550	5.83	2215	10.16
3.780	9315	2.30	1159	5.31
3.980	4820	1.19	466	2.14
4.473	112	0.03	22	0.10
4.987	3675	0.91	421	1.93
5.327	246	0.06	13	0.06
5.707	142	0.04	16	0.07
5.987	3589	0.89	377	1.73

6.260	49	0.01	11	0.05
6.467	52	0.01	22	0.10
6.873	31	0.01	15	0.07
6.933	74	0.02	14	0.06
7.087	107	0.03	20	0.09
7.213	139	0.03	19	0.09
7.380	313	0.08	26	0.12
7.580	185	0.05	26	0.12
7.793	136	0.03	19	0.09
Totals	404288	100.00	21810	100.00
