# Supplementary material for: Intranasal Mucoadhesive In Situ Gel of Glibenclamide-Loaded Bilosomes for Enhanced Therapeutic Drug Delivery to the Brain
Source: Pharmaceutics. 2025 Feb 4;17(2):193. doi: 10.3390/pharmaceutics17020193 (PMC11859129; doi:10.3390/pharmaceutics17020193)
Supplement: Supplementary file 1 [file pharmaceutics-17-00193-s001.zip › Supplementary Table 1.pdf]

Table S1. Mathematical model fitting values obtained from percent drug release profile of glibenclamide loaded bilosomes (F1-F17)

Formulation	Zero order R^2	First order R^2	Higuchi model R^2	Korsmeyer &Peppas(n)	Hixson Crowell R^2	Release order
F1	0.4908	0.8907	0.9747	0.456	0.8498	Higuchi
F2	0.4756	0.7721	0.9212	0.454	0.7397	Higuchi
F3	-2.8865	0.9681	0.0567	0.220	0.6314	First order
F4	0.9326	0.8999	0.9807	0.395	0.9244	Higuchi
F5	0.3492	0.8292	0.9384	0.426	0.7697	Higuchi
F6	0.5536	0.8013	0.9367	0.476	0.7687	Higuchi
F7	0.5589	0.8969	0.9647	0.479	0.8540	Higuchi
F8	0.4799	0.7404	0.9022	0.457	0.7036	Higuchi
F9	0.3492	0.8292	0.9384	0.426	0.7697	Higuchi
F10	0.3476	0.8292	0.9384	0.426	0.7697	Higuchi
F11	-0.1362	0.7084	0.8933	0.353	0.5635	Higuchi
F12	0.4592	0.8376	0.9583	0.447	0.7953	Higuchi
F13	0.1206	-0.2535	0.8565	0.380	0.4881	Higuchi
F14	-1.3690	0.6995	0.5513	0.271	0.5406	First order
F15	-1.6751	0.7270	0.5037	0.261	0.5146	First order
F16	-0.9496	0.5836	0.6135	0.288	0.4516	Higuchi
F17	0.3493	0.8292	0.9384	0.426	0.7697	Higuchi
