# Supplementary figures and images for: Intranasal Mucoadhesive In Situ Gel of Glibenclamide-Loaded Bilosomes for Enhanced Therapeutic Drug Delivery to the Brain
Source: Pharmaceutics. 2025 Feb 4;17(2):193. doi: 10.3390/pharmaceutics17020193 (PMC11859129; doi:10.3390/pharmaceutics17020193)

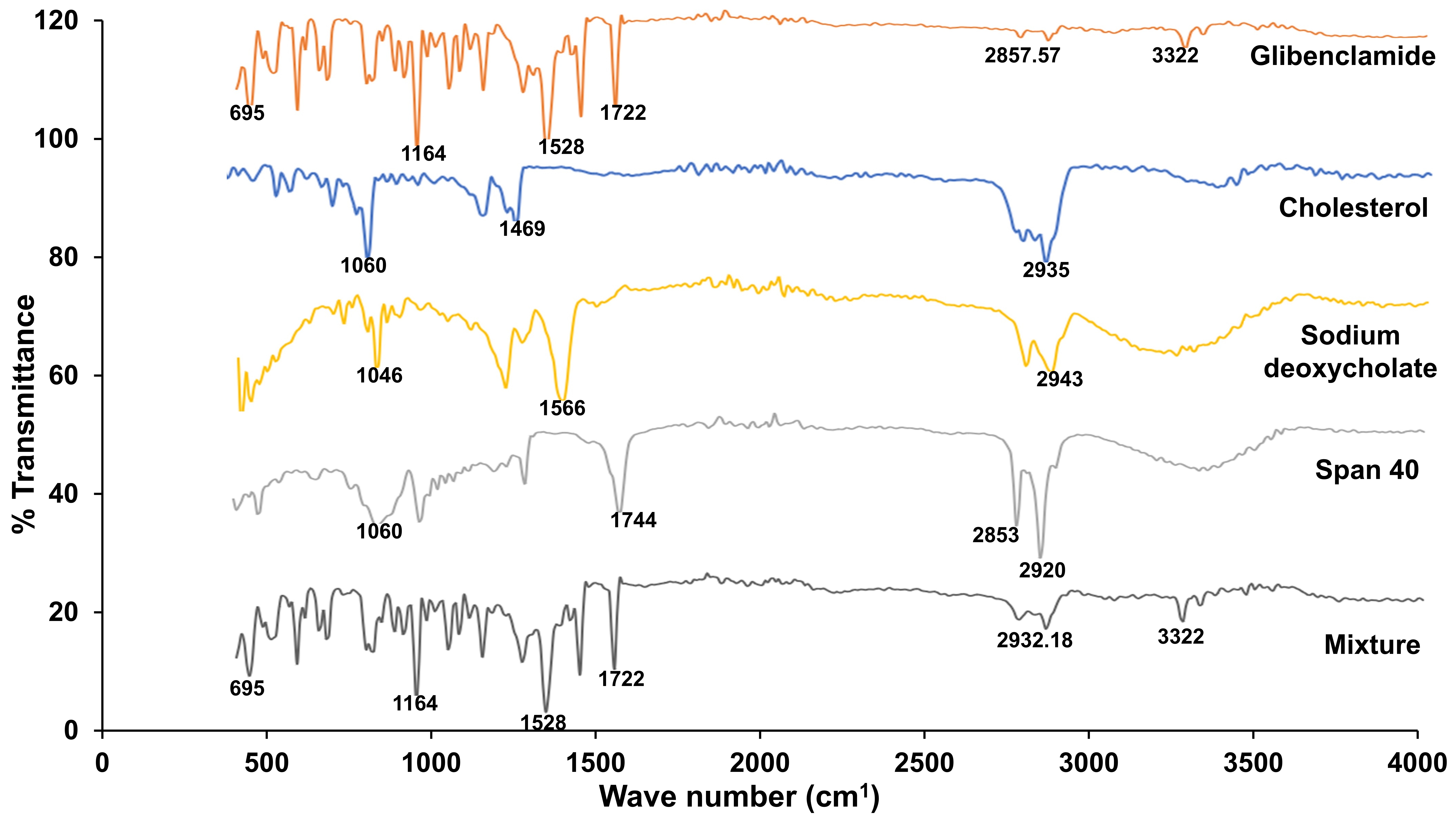

Supplement: Supplementary file 1 [file pharmaceutics-17-00193-s001.zip › Supplementary Figure S1.jpg]
